# Ancestral Physical Stress and Later Immune Gene Family Expansions Shaped Bivalve Mollusc Evolution

**DOI:** 10.1093/gbe/evab177

**Published:** 2021-08-03

**Authors:** Tim Regan, Lewis Stevens, Carolina Peñaloza, Ross D Houston, Diego Robledo, Tim P Bean

**Affiliations:** 1 The Roslin Institute and Royal (Dick) School of Veterinary Studies, University of Edinburgh, United Kingdom; 2 Tree of Life Programme, Wellcome Sanger Institute, Wellcome Genome Campus, Hinxton, Cambridge, United Kingdom

**Keywords:** bivalve, mollusc, orthology, evolution

## Abstract

Bivalve molluscs comprise 20,000 species occupying a wide diversity of marine habitats. As filter feeders and detritivores they act as ecosystem engineers clarifying water, creating reefs, and protecting coastlines. The global decline of natural oyster reefs has led to increased restoration efforts in recent years. Bivalves also play an important role in global food security contributing to >20% of worldwide aquaculture production. Despite this importance, relatively little is known about bivalve evolutionary adaptation strategies. Difficulties previously associated with highly heterozygous and repetitive regions of bivalve genomes have been overcome by long-read sequencing, enabling the generation of accurate bivalve assemblies. With these resources we have analyzed the genomes of 32 species representing each molluscan class, including 15 bivalve species, to identify gene families that have undergone expansion during bivalve evolution. Gene family expansions across bivalve genomes occur at the point of evolutionary pressures. We uncovered two key factors that shape bivalve evolutionary history: expansion of bivalvia into environmental niches with high stress followed by later exposure to specific pathogenic pressures. The conserved expansion of protein recycling gene families we found across bivalvia is mirrored by adaptations to a sedentary lifestyle seen in plants. These results reflect the ability of bivalves to tolerate high levels of environmental stress and constant exposure to pathogens as filter feeders. The increasing availability of accurate genome assemblies will provide greater resolution to these analyses allowing further points of evolutionary pressure to become clear in other understudied taxa and potentially different populations of a single species.

## Main Text

Bivalves are ecosystem engineers. Through filter-feeding they recycle nutrients, clarify water, and can protect coastlines from extreme weather by reef formation (Helmer et al. 2019; van der Schatte Olivier et al. 2020; Ray and Fulweiler 2021). The ongoing global decline of wild oyster reefs has led to an interest in applying restorative aquaculture to recover these vital ecosystem services (Vaughn and Hoellein 2018). They also comprise >20% of global aquaculture production (FAO 2020; Houston et al. 2020). To establish a community for restocking or aquaculture, robust stocks are crucial, underscoring the importance of effective breeding strategies (Gutierrez et al. 2017; Potts et al. 2021) which in turn require better understanding of immunity and resilience mechanisms employed by bivalves (Tan et al. 2020).

Since diverging from other molluscs approximately 530 Ma (Kocot et al. 2020), bivalves have adapted to a diverse range of niches including freshwater, intertidal zones, abyssal plains, and deep sea hydrothermal vents (Vaughn and Hoellein 2018), with over 20,000 known species globally. However, we know relatively little about what has allowed bivalves to thrive in these diverse habitats. Due to their largely sedentary or sessile lifestyle (Vaughn and Hoellein 2018), parallels have been drawn between bivalves and long-lived, highly fecund plants (Williams 1975; Plough 2016). Sessility requires adaptation to local environmental stressors such as air exposure and variations in temperature, pH, and salinity, whereas filter-feeding exposes bivalves to a wide range of chemical and pathogenic stressors in the water column (Burge et al. 2016). This constant exposure requires robust adaptation mechanisms, and the molecular basis of bivalve stress responses and the gene families involved deserves further attention.

Exploring the evolution of these gene families requires high-quality bivalve reference genomes. Generating such genomes has been impeded by high levels of heterozygosity (Plough 2016; Takeuchi 2017; Hollenbeck and Johnston 2018), repetitive regions (Davison and Neiman 2021), and structural variations (Calcino et al. 2021). A pan-genome based on gene presence–absence variation has recently been suggested for the Mediterranean mussel (Gerdol et al. 2020). However, long-read sequencing (Sun et al. 2021) has somewhat overcome these issues, leading to an increasing number of improved assemblies (Caurcel et al. 2021; Peñaloza et al. 2021). These resources present a new opportunity; here, using the genomes of 32 species representing each molluscan class, including 15 bivalve species, we identify gene families which have expanded during early and recent bivalve evolution.

**Fig. 1.**
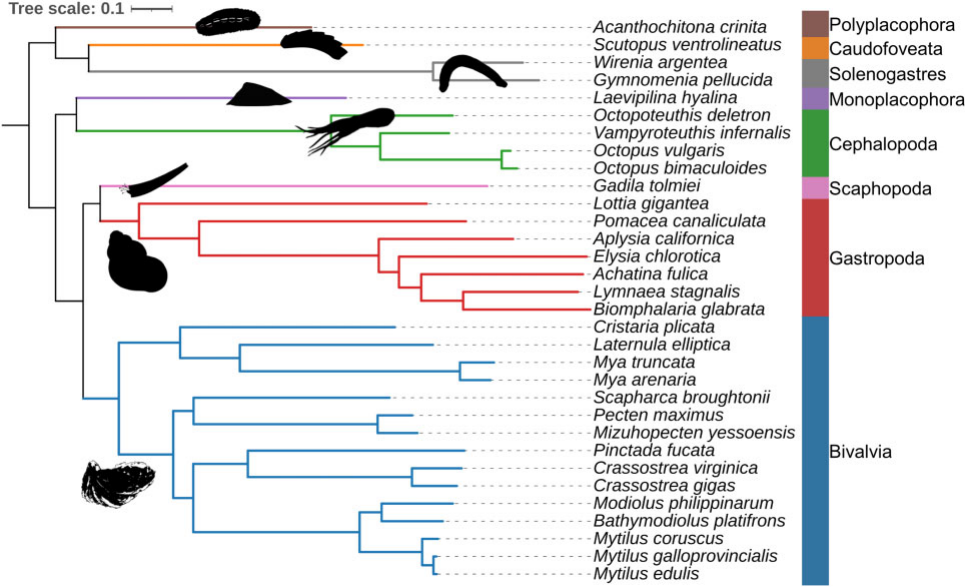
The molluscan phylogeny. Branch lengths are in amino acid substitutions per site; scale is shown. Colors on tree branches represent each of the molluscan classes as indicated. Silhouettes are reproduced from http://phylopic.org.

Using OrthoFinder (Emms and Kelly 2019), we clustered the longest isoform of each protein-coding gene from each species into orthogroups (OGs), that is, orthologous groups of genes sharing a common ancestor. This resulted in >90% of all proteins being assigned to an OG (supplementary fig. 2, Supplementary Material online). We inferred the molluscan phylogeny using a concatenated alignment of 813 single-copy orthologs present in at least 28 of the 32 species and maximum likelihood under the general-time reversible substitution model with gamma-distributed rate variation among sites (GTR + Γ). The phylogeny is rooted on the branch separating Aculifera (a clade comprising Caudofoveata, Polyplacophora, and Solenogastres) from Conchifera (a clade comprising Bivalvia, Cephalopoda, Gastropoda, Monoplacophora, and Scaphopoda). The resulting topology was congruent with previously published phylogenies and all molluscan classes were recovered as monophyletic (Kocot et al. 2020) (fig. 1).

**Fig. 2.**
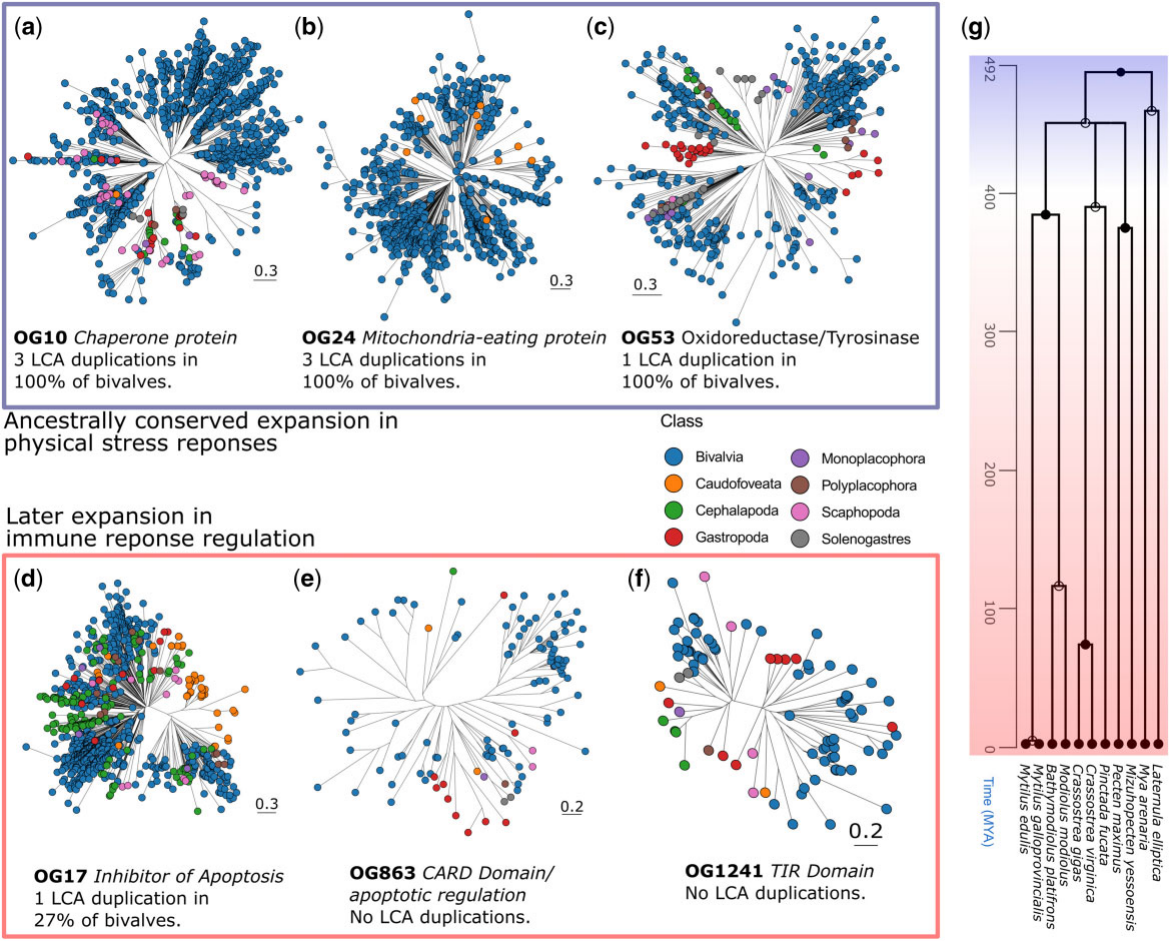
Gene families that have undergone expansion in bivalves. Trees of gene families with duplication events conserved in 100% of bivalves since LCA (*a*–*c*) and more recent expansion (*d*–*f*). Timescale phylogeny tree for bivalves (*g*) produced using TimeTree (Kumar et al. 2017). Gene family name and function are displayed with number of LCA duplications conserved across all bivalves. Gene trees were inferred using FastTree (Price et al. 2010) within OrthoFinder and plotted using ggtree (Yu et al. 2017). Nodes representing genes are colored according to taxonomic class.

We used two complementary approaches to identify gene families that have undergone expansion in bivalves. First, using the molluscan species tree and OrthoFinder, we inferred gene trees and gene duplication events. Of these, we looked at OGs with an arbitrarily designated minimum of 5-fold genes per species in bivalves relative to other molluscs which also contained duplications that occurred in the last common ancestor (LCA) of all bivalve taxa and that have been retained by >70% of all bivalve species in our study. The aim of this analysis was to focus on OGs with more genes in bivalves relative to other molluscs which also contained conserved duplication events. Second, we used KinFin (Laetsch and Blaxter 2017) to identify gene families that were significantly (*P* value <4×10^−5^) overrepresented in bivalves relative to other molluscan taxa (referred to as “bivalve-enriched”), regardless of presence in LCA. This latter approach allowed us to identify gene families that have undergone expansion in bivalves over ancient and also more recent evolutionary time scales (i.e., clade- or species-specific expansions). In total, we identified 16 gene families with ancestrally conserved duplications across all bivalves (table 1; examples shown in fig. 2*a–c*) and 15 that were significantly bivalve-enriched (table 2; examples shown in fig. 2*d–f*). Two gene families, OG10 and OG53, were found in both analyses. We also analyzed the genome-wide distribution of these expanded gene families by comparing their positions across five chromosomally resolved molluscan reference genomes (supplementary fig. 4, Supplementary Material online).

**Table 1:** OGs with Conserved Ancestral Duplications and a Relatively High Number of Genes per Species in Bivalves

Orthogroup		Avg. Genes/Species		No. of Duplications in LCA Retained by >70% of Bivalves	Functional Annotation	Source
Name	Size	Bivalvia (15)	Others (17)			
OG643	143	9.5	0	1	Domain ATPase, dynein-related, AAA domain Negative regulation of noncanonical Wnt signaling pathway	IPR, GO, Pfam eggNOG
OG950	110	7.3	0	1	—	—
OG1538	82	5.5	0		OTU-like cysteine protease Thiol-dependent ubiquitin-specific protease activity	IPR, Pfam eggNOG
OG2450	63	4.2	0	1	—	—
OG3835	50	3.3	0	1	Domain of unknown function (DUF1772)	Pfam, eggNOG
OG92	441	29.3	0.1	1	Complement C1q domain	IPR, Pfam, eggNOG
OG24	807	53.1	0.6	3 (3 that are conserved in 100% of bivalves)	Mitochondria-eating protein	IPR, Pfam
OG708	135	8.9	0.1	1	Domain B-box-type zinc finger Interferon-beta production	IPR, GO, Pfam eggNOG
OG314	234	15.1	0.4	1	Mib-herc2 Protein ubiquitination	IPR, Pfam GO, eggNOG
OG1139	99	6.4	0.2	2	Zinc finger, C3HC4 type (RING finger)	IPR, GO, Pfam, eggNOG
OG913	113	7.1	0.4	1	Repeat leucine-rich repeat	IPR, Pfam, eggNOG
OG10	1,096	68.1	4.4	8 (3 that are conserved in 100% of bivalves)	Heat shock protein 70 family Regulation of apoptotic process	Pfam, IPR, eggNOG GO
OG3669	51	2.9	0.4	1	—	—
OG888	115	6.5	1.1	1	Rho GTPase	IPR, GO, Pfam, eggNOG
OG53	552	30.6	5.5	3 (1 that is conserved in 100% of bivalves)	Tyrosinase copper-binding domain Oxidoreductase activity	IPR, Pfam, eggNOG GO
OG29	767	41.8	8.2	1	Neuronal acetylcholine receptor subunit	IPR, GO, Pfam, eggNOG

Note.—OGs with at least five times as many bivalve genes per species compared with other molluscan classes, and at least one duplication event in LCA of bivalves retained by >70% of bivalve species used in this study.

**Table 2:** Functional Annotation of Bivalve-Enriched Orthogroups (each bivalve species used has at least one gene, cut-off *P* value <4×10^−5^)

Orthogroup		Avg. Genes/Species		No. of Duplications in LCA Retained by >50% of Bivalves	*P* Value (bivalvia vs. others)	Functional Annotation	Source
Name	Size	Bivalves (15)	Others (17)				
OG3213	54	2.4	1.1	1	4.48E-07	Domain F-box domain Regulation of muscle adaptation	IPR, Pfam eggNOG
OG10	1,096	68.1	4.4	11	1.27E-06	Heat shock protein 70 family Regulation of apoptotic process	Pfam, IPR, eggNOG GO
OG1241	93	4.7	1.3	0	2.17E-06	Toll/interleukin-1 receptor homology (TIR) domain	Pfam, IPR, eggNOG
OG863	117	6.5	1.1	0	5.01E-06	CARD domain Regulation of apoptotic process	IPR, Pfam, eggNOG GO
OG13	949	56.5	5.9	1	7.07E-06	Complement C1q domain	IPR, Pfam, eggNOG
OG53	552	30.6	5.5	3	7.87E-06	Tyrosinase copper-binding domain Oxidoreductase activity	IPR, Pfam, eggNOG GO
OG268	257	13.1	3.6	0	9.12E-06	Domain Deltex, C-terminal E3 ubiquitin-protein ligase	IPR, Pfam eggNOG
OG272	255	15.1	1.7	1	9.54E-06	Repeat LDLR class B repeat Endocytosis	IPR, Pfam eggNOG
OG298	240	13.7	2.0	0	1.12E-05	Galactose-binding lectin domain Autophagy and apoptosis	IPR, Pfam eggNOG
OG4321	47	2.0	1.0	0	1.37E-05	Phosphatase activity	Pfam, GO, IPR
OG17	919	46.4	13.1	0	1.76E-05	Inhibitor of Apoptosis domain	Pfam, IPR, eggNOG
OG498	171	9.2	1.9	0	2.78E-05	TROVE domain U2 snRNA binding	IPR, Pfam eggNOG
OG18	905	38.2	19.5	0	3.11E-05	EF-hand domain Calmodulin	IPR, Pfam GO, eggNOG
OG857	117	5.7	1.8	0	3.33E-05	Acyl-CoA oxidase, C-terminal	Pfam, IPR, GO, eggNOG
OG2	2,007	102.7	27.4	0	3.82E-05	C-type lectin-like	Pfam, IPR, eggNOG

The largest heat shock protein (HSP) family identified from our analysis (OG10, the tenth largest molluscan OG overall) (fig. 2*a*) was the second most significantly bivalve-enriched OG (*P *=* *1.27×10^−6^, table 2). This is consistent with the previous genomics and molecular biology studies highlighting the significance of HSPs in bivalves for maintaining cellular function during exogenous redox/chemical/pH stress. Bivalves may also induce intracellular stress to handle constant exposure to intracellular pathogens, requiring chaperone and HSPs to prevent apoptosis and retain cellular function (Venier et al. 2011; Gust et al. 2013; Leite et al. 2013; Al-Khalaifah and Afaf 2018; Smits et al. 2020). In bivalves, members of this gene family are distributed throughout the genome (e.g., in *Crassostrea gigas*, they are present on six of the ten chromosomes; supplementary fig. 4*a* and *b*, Supplementary Material online), whereas they are restricted to two chromosomes in the gastropod *Pomacea canaliculata* (supplementary fig. 4*c*, Supplementary Material online) and one chromosome in the cephalopod *Octopus vulgaris* (supplementary fig. 4*d*, Supplementary Material online). Mitochondria-eating protein (*Mieap*) families are responsible for maintaining healthy mitochondria after intracellular damage (Kitamura et al. 2011; Nakamura and Arakawa 2017). The largest *Mieap* OG identified in our analysis was OG24 (fig. 2*b*). These OGs (fig. 2*a–c*) each had three duplications in the LCA conserved across all bivalves used in this analysis (table 1). Mitophagy results in high levels of intracellular reactive oxygen species, requiring chaperone proteins to maintain cellular functioning (Rose et al. 2011). The fact that both of these large gene families operate to regulate intracellular damage suggests that this was an important function during early bivalve evolution.

Hypoxic stress is inextricable from bivalve life history in the intertidal environment where access to oxygenated water is dependent on tide. It is also a secondary effect of the primary defence mechanism of shell clamping (Long et al. 2008) and is induced by bivalves during infection (Al-Khalaifah and Afaf 2018; Smits et al. 2020; Steffen et al. 2020). OG53, the second most significantly bivalve-enriched gene family, consists of oxidoreductase and tyrosinase proteins (*P *=* *7.87×10^−6^, table 2). This OG contained one duplication event in the LCA of all bivalves that has since been retained by all bivalve species we sampled (fig. 2*c*). In addition to hypoxic stress, this gene family may also be involved in melanization (Kobayashi et al. 1994), a well described invertebrate wound response (Bilandzija et al. 2017) and another physical stress response.

As bivalves lack an adaptive immune system, constant pathogen exposure necessitates specialized tolerance mechanisms (Wang et al. 2013). Protein recycling pathways, chaperone proteins, and apoptotic inhibitors can be employed to maintain cellular function during infection by inducing high levels of oxidative stress to remove intracellular pathogens (Sunila and LaBanca 2003; Donaghy et al. 2009; Hughes et al. 2010; Zhang et al. 2011; Brulle et al. 2012; McDowell et al. 2014; Smits et al. 2020). This is reflected in the high diversity of inhibitor of apoptosis proteins (IAPs) shared by bivalves (Song et al. 2021; Vogeler et al. 2021). The largest IAP gene family we identified was OG17. Although this OG contained 3-fold more duplications in bivalves compared with other molluscan classes (table 2), these duplications were not ancestrally retained across Bivalvia (no LCA duplications retained by >50% of bivalves used in this study), suggesting more recent expansion and diversification (fig. 2*d*). This may reflect selective pressure from certain pathogens that can take advantage of these pathways, for example, during *Bonamia ostreae* and OsHV-1 infections (de Lorgeril et al. 2018; Cocci et al. 2020).

Central to initiation of the innate immune transcriptional response is inflammasome formation, largely regulated by IAP and CARD protein families (Latz et al. 2013). The fourth most significantly bivalve-enriched OG (*P *=* *5.01×10^−6^) was a CARD protein family (OG863, table 2). Similar to the IAP gene family, no LCA duplication events were found in this OG suggesting more recent expansion and diversification (fig. 2*e*). The earliest phase of TLR signal transduction following pathogen detection is regulated by Toll/interleukin-1 receptor homology (TIR) domain-containing proteins (O’Neill and Bowie 2007). OG1241, a TIR domain protein family, was the third most significantly bivalve-enriched OG (*P *=* *2.17×10^−6^) with an average of 3-fold more copies in bivalve species compared with nonbivalve molluscs (table 2). Again, this gene family contained no LCA duplication events across bivalves (fig. 2*f*).

Unlike other OGs with associated immune function, the *C1q* gene family OG92 contained a LCA duplication event retained by >70% of bivalves. OG92 also consisted almost entirely of bivalve genes and is likely to have expanded greatly over time following the ancestral duplication event. Although the innate immune complement system is best described role for *C1q*, its function has diversified over time with recently described shell formation proteins described in bivalves for this protein family (Xiong et al. 2021). This presents an example of LCA duplications in an OG retained across bivalves followed by further expansions over time and potential caveats of ascribing single functions to entire gene families based on orthology.

Similar to plants, bivalves are mostly sessile and require the ability to respond to highly transient environmental conditions in a rapid and efficient manner. Protein recycling gene family expansion in plants is thought to be an adaptation to sessile lifestyle with protein modifications enabling a fast and easily reversible modulation of protein function (Hua and Vierstra 2011; Park and Seo 2015; Chi et al. 2019). It is possible that bivalves have evolved similar mechanisms to adapt to sessility. We identified multiple protein recycling gene families that were overrepresented in bivalves (tables 1 and 2) including two OGs unique to Bivalvia (OG643, OG1538). In addition to informing lifestyle adaptation, this may underscore the importance of proteomics in experiments investigating bivalve resilience/immune mechanisms. For instance, proteomics revealed the importance of redox homeostasis for resistance to brown ring disease in a Manilla clam infection challenge (Smits et al. 2020).

Expansion of complement, redox, chaperone, and protein recycling enzyme families across bivalves is thought to have occurred in a species-specific manner (Wang et al. 2010, 2016; Fleury and Huvet 2012; Takeuchi et al. 2016). We found evidence of conserved ancestral duplication (retained by >66% of bivalves) among four gene families (OG92, OG10, OG53, OG314, and OG1538) (table 1). In the context of innate immunity, pattern recognition receptors are described as having undergone a broad expansion across molluscan evolution (Vasta and Wang 2020). We found gene families with immune function, that is, TLR, CLR, and inflammasome regulation (OG2, OG17, OG863, and OG1241) underwent more recent expansion with little or no conserved LCA duplications (table 2). This could reflect the fact that all or most bivalves experience similar physiological stressors from fluctuations in temperature, pH, tides, and oxygen availability. Although these stressors have remained constant since the LCA of bivalves, pathogenic challenges continue to evolve over time requiring more recent adaptations (Obbard et al. 2009; Webb et al. 2015). This is reflected in the clade- and species-specific diversification among gene families regulating innate immunity.

Using genome biology to examine evolutionary history of gene families, we have revealed and differentiated both recent and ancestral adaptation strategies. Future studies using different populations of bivalves should further explore the gene families described here to identify their redundancies and functional diversity. This improved understanding of basic biology will provide fundamental knowledge which can aid bivalve aquaculture or restoration efforts. We have primarily focused on OGs containing more genes in bivalves relative to other molluscs on the assumption that expansion of gene families across this class correlate with natural selection. It should be noted that neutral forces as well as selective forces may lead to such expansion and these forces should also be analyzed further in future studies (Hahn et al. 2005).

With the rapidly increasing availability and improvement of genome assemblies, the methods described here will allow for more specific biological analyses, including higher taxonomic resolution. For instance, by adopting a similar approach, studies examining adaptation of different populations across a single species could focus on gene families across the taxonomic class with and without conserved duplications, using sister species to define the LCA, rather than species from a different taxonomic class. This demonstrates the utility of genome biology to better understand the evolutionary history of understudied species.

## Materials and Methods

### Orthology Clustering

Details of the proteomes used in our analyses are detailed in supplementary table 1, Supplementary Material online. Briefly, we selected the longest isoform for each protein-coding gene in each species using AGAT (version 0.4.0) (Jacques Dainat 2020). We assessed the completeness and level of duplication in each of the isoform-filtered proteomes using BUSCO (version 4.06 against mollusca_odb10 database) (Seppey et al. 2019) and CD-hit (version 4.6.8) (Fu et al. 2012) using 90% sequence identity threshold and a word length of 5 (supplementary fig. 1, Supplementary Material online). We clustered the isoform-filtered proteomes into OGs using OrthoFinder (version 2.3.11) (Emms and Kelly 2019).

### Species Tree Inference

A very small number of OGs (3 in total) were single copy across all 32 species, likely as result of incomplete assemblies and/or annotations or as a result of haplotypic duplication. To circumvent this, we selected 2,933 OGs that were present in at least 75% of species and had an average count of 1 using KinFin (v1.0; Laetsch and Blaxter 2017). To remove clusters containing paralogous sequences, we aligned the protein sequences of each selected OG using MAFFT (v7.455; Katoh and Standley 2013) and generated a maximum likelihood tree along with 1,000 ultrafast bootstraps (Hoang et al. 2018) using IQ-TREE (v2.0.3; Nguyen et al. 2015), allowing the best-fitting substitution model to be selected automatically (Kalyaanamoorthy et al. 2017). We screened each gene tree for evidence of paralogy using PhyloTreePruner (v1.0; Kocot et al. 2013) and retained 813 OGs containing orthologous sequences from at least 28 of the 32 species. If two representative sequences were present for any species (i.e., in-paralogs) after this paralog screening step, the longest of the two sequences was retained and the other discarded. We realigned the protein sequences of each filtered OG using MAFFT and trimmed spuriously aligned regions from each alignment using trimAl. The trimmed alignments were concatenated using catfasta2phyml (available from: https://github.com/nylander/catfasta2phyml) to form a supermatrix. We inferred the species tree using maximum likelihood using IQ-TREE, with the general-time reversible (GTR) substitution model with gamma-distributed rate variation among sites (+Γ) along with 1,000 ultrafast bootstraps. As per Kocot et al. (2013), we rooted the resulting phylogeny on the branch separating the aculiferans (a clade comprising Caudofoveata, Polyplacophora, and Solenogastres) from the conchiferans (a clade comprising Bivalvia, Cephalopoda, Gastropoda, Monoplacophora, and Scaphopoda). The phylogeny was visualized using iToL (available at https://itol.embl.de/).

### Identification of Expanded Gene Families

To identify gene families that underwent expansion during early bivalve evolution, we provided the orthology clustering and the inferred species tree to OrthoFinder, which inferred gene duplication events. Briefly, OrthoFinder infers gene trees for each OG using FastTree and uses the rooted species tree to infer gene duplication events using duplication-loss-coalescent model. We identified OGs that underwent gene duplication events in the LCA of all bivalve species and selected only those that had been retained by 11 of 15 bivalve species in our study, as an arbitrarily designated cutoff. Of these, we kept OGs where bivalves had an average of five times as many genes than species from other molluscan classes (table 1). To identify gene families that have undergone expansion during more recent bivalve evolution, we used KinFin (version 1.0) to identify OGs that were significantly overrepresented in bivalves compared with nonbivalve molluscs. We selected OGs that had a *P* value <4×10^−5^ (table 2). For both sets of expanded OGs, we visualized the gene tree used ggtree R package (Yu et al. 2017). We also functionally annotated each expanded OG by searching each protein against the Pfam (Mistry et al. 2021) database and eukaryotic SignalP database (Petersen et al. 2011) using InterProScan (version 5.47-82.0) (Jones et al. 2014) and provided the resulting annotations to KinFin (Laetsch and Blaxter 2017) or using the eggNOG mapper (http://eggnog-mapper.embl.de/) (Huerta-Cepas et al. 2017).

### Synteny

To analyze chromosome rearrangements and synteny between bivalves and other molluscs, we identified all one-to-one orthologs between *C. gigas* and each of the four assemblies used *Crassostrea virginica*, *Pecten maximus*, *Pomacea canaliculata*, or *Octopus vulgaris*. We then mapped the locations of each gene from selected bivalve paralog-rich OGs with recent, or ancestrally conserved duplications (OG10 and OG1241). Circos plots were generated using Circos v0.69–8 (Krzywinski et al. 2009).

## Supplementary Material

Supplementary data are available at *Genome Biology and Evolution* online.

## Acknowledgments

This work was supported by funding from the Biotechnology and Biological Sciences Research Council (BBS/E/D/10002070, BBS/E/D/30002275, BBS/E/D/10002071) and the Wellcome Trust (206194, 218328).

## Data Availability

Data relevant to this study including the genome assemblies used, supermatrix file of the concatenated alignments of 813 single-copy orthologs used to infer the species tree, orthology clustering file, the species tree used to infer duplications, orthology clustering file of protein sequences and gene duplications from Orthofinder and KinFin results are available in Zenodo with the identifier “doi:10.5281/zenodo.4697197.”

## Supplementary Material

evab177_Supplementary_Data
