# Supplementary material for: Ancestral Physical Stress and Later Immune Gene Family Expansions Shaped Bivalve Mollusc Evolution
Source: Genome Biol Evol. 2021 Aug 3;13(8):evab177. doi: 10.1093/gbe/evab177 (PMC8382680; doi:10.1093/gbe/evab177)
Supplement: evab177_Supplementary_Data [file evab177_supplementary_data.docx]

**Supplementary Data**

**Supplementary Table 1** Assemblies used (name of species with accompanying assembly)

| **Class** | **Species** | **Source** | **Data url** |
| --- | --- | --- | --- |
| Bivalvia | *Bathymodiolus platifrons* | MDB | <https://download.molluscdb.org/v2/> |
|  | *Crassostrea gigas* | NCBI | <https://ftp.ncbi.nlm.nih.gov/genomes/all/GCF/902/806/645/GCF_902806645.1_cgigas_uk_roslin_v1/GCF_902806645.1_cgigas_uk_roslin_v1_protein.faa.gz> |
|  | *Crassostrea virginica* | NCBI | <https://ftp.ncbi.nlm.nih.gov/genomes/all/GCF/002/022/765/GCF_002022765.2_C_virginica-3.0/GCF_002022765.2_C_virginica-3.0_protein.faa.gz> |
|  | *Cristaria plicata* | MDB | <https://download.molluscdb.org/v2/> |
|  | *Laternula elliptica* | MDB | <https://download.molluscdb.org/v2/> |
|  | *Mizuhopecten yessoensis* | NCBI | <https://ftp.ncbi.nlm.nih.gov/genomes/all/GCF/002/113/885/GCF_002113885.1_ASM211388v2/GCF_002113885.1_ASM211388v2_protein.faa.gz> |
|  | *Modiolus philippinarum* | MDB | <https://download.molluscdb.org/v2/> |
|  | *Mya arenaria* | MDB | <https://download.molluscdb.org/v2/> |
|  | *Mya truncata* | MDB | <https://download.molluscdb.org/v2/> |
|  | *Mytilus coruscus* | NCBI | <https://ftp.ncbi.nlm.nih.gov/genomes/all/GCA/011/752/425/GCA_011752425.2_MCOR1.1/GCA_011752425.2_MCOR1.1_protein.faa.gz> |
|  | *Mytilus edulis* | MDB | <https://download.molluscdb.org/v2/> |
|  | *Mytilus galloprovincialis* | MDB | <https://download.molluscdb.org/v2/> |
|  | *Pecten maximus* | NCBI | <https://ftp.ncbi.nlm.nih.gov/genomes/all/GCF/902/652/985/GCF_902652985.1_xPecMax1.1/GCF_902652985.1_xPecMax1.1_protein.faa.gz> |
|  | *Pinctada fucata* | OIST Marine Genomics Unit | <https://marinegenomics.oist.jp/pearl/viewer?project_id=36> |
|  | *Scapharca broughtonii* | GigaDB | <ftp://parrot.genomics.cn/gigadb/pub/10.5524/100001_101000/100607/EVM.final.gene.gff3.pep> |
| Caudofoveata | *Scutopus ventrolineatus* | MDB | <https://download.molluscdb.org/v2/> |
| Cephalapoda | *Octopoteuthis deletron* | MDB | <https://download.molluscdb.org/v2/> |
|  | *Octopus bimaculoides* | NCBI | <https://ftp.ncbi.nlm.nih.gov/genomes/all/GCF/001/194/135/GCF_001194135.1_Octopus_bimaculoides_v2_0/GCF_001194135.1_Octopus_bimaculoides_v2_0_protein.faa.gz> |
|  | *Octopus vulgaris* | NCBI | <https://ftp.ncbi.nlm.nih.gov/genomes/all/GCF/006/345/805/GCF_006345805.1_ASM634580v1/GCF_006345805.1_ASM634580v1_protein.faa.gz> |
|  | *Vampyroteuthis infernalis* | MDB | <https://download.molluscdb.org/v2/> |
| Gastropoda | *Achatina fulica* | GigaDB | <http://gigadb.org/dataset/100647> |
|  | *Aplysia californica* | NCBI | <https://ftp.ncbi.nlm.nih.gov/genomes/all/GCF/000/002/075/GCF_000002075.1_AplCal3.0/GCF_000002075.1_AplCal3.0_protein.faa.gz> |
|  | *Biomphalaria glabrata* | NCBI | <https://ftp.ncbi.nlm.nih.gov/genomes/all/GCF/000/457/365/GCF_000457365.1_ASM45736v1/GCF_000457365.1_ASM45736v1_protein.faa.gz> |
|  | *Elysia chlorotica* | NCBI | <https://ftp.ncbi.nlm.nih.gov/genomes/all/GCA/003/991/915/GCA_003991915.1_ElyChl2.0/GCA_003991915.1_ElyChl2.0_protein.faa.gz> |
|  | *Lottia gigantea* | MDB | <https://download.molluscdb.org/v2/> |
|  | *Lymnaea stagnalis* | MDB | <https://download.molluscdb.org/v2/> |
|  | *Pomacea canaliculata* | NCBI | <https://ftp.ncbi.nlm.nih.gov/genomes/all/GCF/003/073/045/GCF_003073045.1_ASM307304v1/GCF_003073045.1_ASM307304v1_protein.faa.gz> |
| Monoplacophora | *Laevipilina hyalina* | MDB | <https://download.molluscdb.org/v2/> |
| Polyplacophora | *Acanthochitona crinita* | MDB | <https://download.molluscdb.org/v2/> |
| Scaphopoda | *Gadila tolmiei* | MDB | <https://download.molluscdb.org/v2/> |
| Solenogastres | *Gymnomenia pellucida* | MDB | <https://download.molluscdb.org/v2/> |
|  | *Wirenia argentea* | MDB | <https://download.molluscdb.org/v2/> |


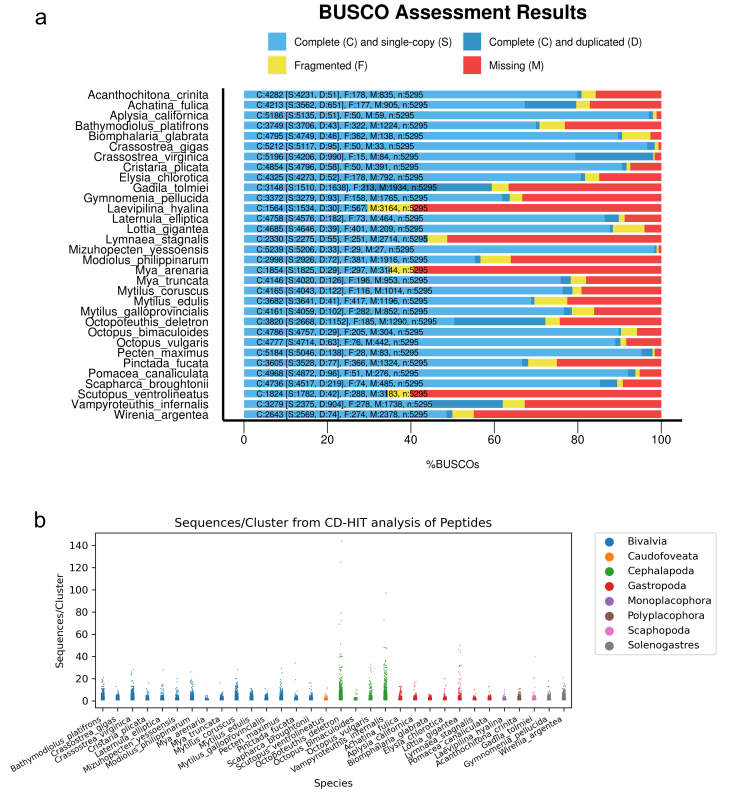


**Supplementary Figure 1: Proteome quality assessment**

(a) BUSCO scores for all 32 species assessed using the ‘mollusca_odb10’ dataset.

(b) CD-Hit analyses of longest-isoform filtered assemblies. The number of sequences per cluster defined by CD-hit using a percentage identity cut off of 90%. Higher numbers per cluster indicate higher proteome redundancy.


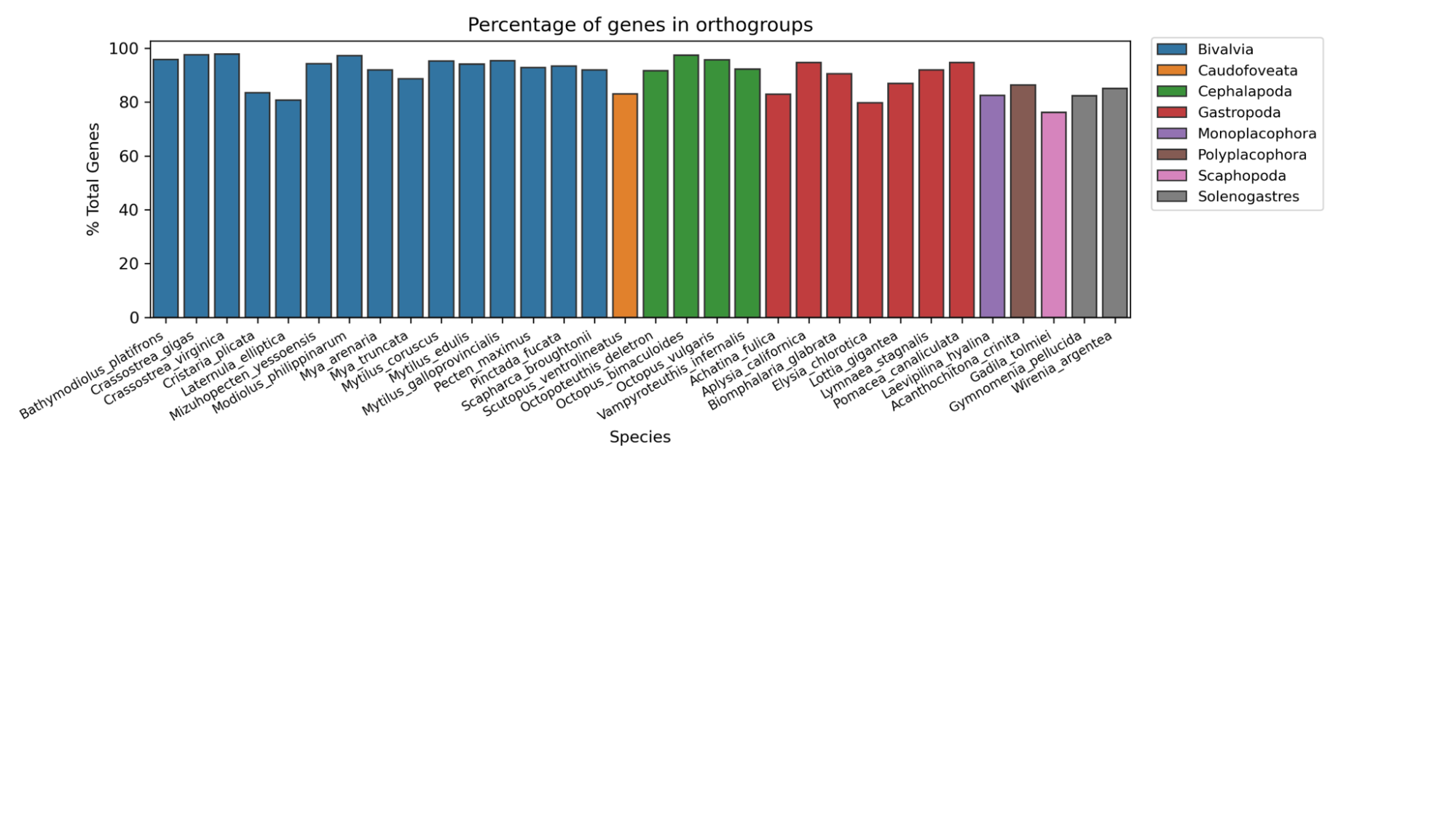


**Supplementary Figure 2: Orthogroup assignment per species**

Percentage of genes from each genome successfully assigned to orthogroups by OrthoFinder.


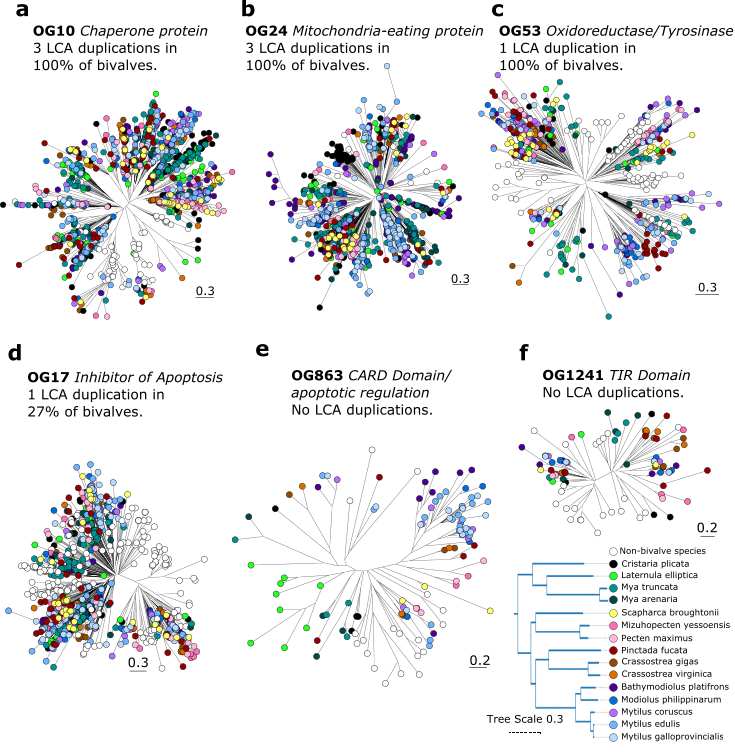


**Supplementary Figure 3** **Mollusc gene families with over-representation of bivalves.** Trees of gene families with duplication events conserved since LCA in 100% of bivalves (a - c) and more recent expansion (d - f). Gene family name and function are displayed with number of LCA duplications conserved across all bivalves. Nodes representing genes are coloured according to bivalve species.


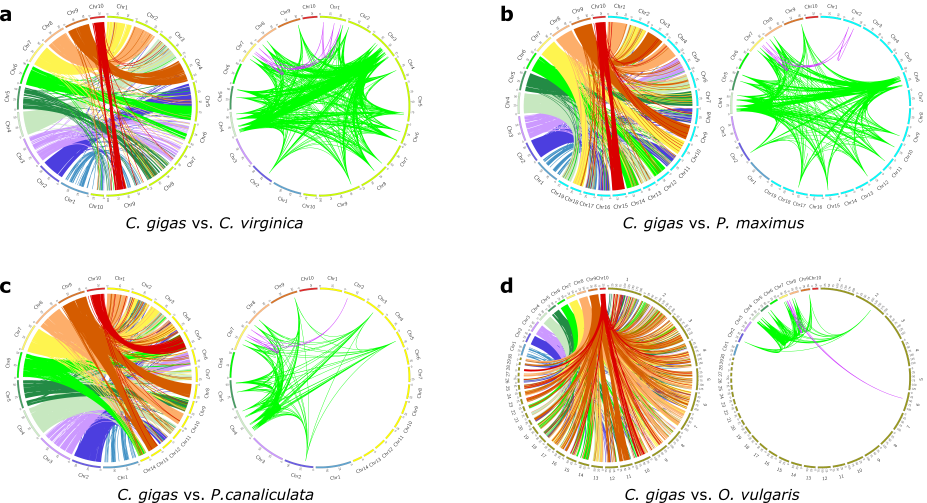
**Supplementary Figure 4** a - d: Pairwise Synteny plots of *Crassostrea gigas* vs. *Crassostrea virginica* (a), *Pecten maximus* (b), *Pomacea canaliculata* (c) and *Octopus vulgaris* (d) for one-to-one orthologues (left) and selected OGs (right). OG10 = green (all bivalves retain at least 3 duplications from LCA of bivalves), OG1241 = purple (no duplications in LCA of bivalves retained). Ticks in each chromosome are in Mb.
